# Retired doctors as users of patient-facing electronic health records: a mixed-methods survey in the UK and Spain

**DOI:** 10.1186/s12911-026-03756-y

**Published:** 2026-08-14

**Authors:** Ray Jones, Angeles Lazcoz, Shangming Zhou, Brian McMillan, Olanrewaju Bamidele, Richard Fitton, Brian Fisher, Mar Soler-Lopez, Charlotte Blease

**Affiliations:** 1 Centre for Health Technology, University of Plymouth, Plymouth, UK; 2 Segovia, Spain; 3 Manchester, UK; 4 London, UK; 5 Plymouth, UK; 6 Division of Population Health, University of Manchester, Manchester, UK; 7 Department of Women’s and Children’s Health, Uppsala University, Box 256, Uppsala, 75105 Sweden

**Keywords:** Online patient access, Retired doctors, UK: Spain, Online survey

## Abstract

**Background:**

Online patient access to electronic records is expanding internationally but remains contested. Although practising clinicians’ concerns are well documented, less is known about the views of retired doctors who are more likely themselves to be patients. We explored the experience of retired doctors in accessing their own medical records as patients in the UK and Spain.

**Methods:**

We conducted an anonymous mixed-methods online survey of retired doctors aged 60 years and over in the UK and Spain. Participants reported their experiences of accessing their own medical records and their views on whether online access should be available to all patients. Quantitative analyses examined factors associated with support; free-text responses were analysed thematically.

**Results:**

Two hundred retired doctors participated (UK n=92; Spain n=108). Overall, 64% supported online access to health records for all patients, with no clear difference between countries. This proportion was higher than that reported in a previous survey of practising general practitioners in England, in which approximately one-third regarded online record access as a good idea; however, the studies involved different populations and sampling approaches and were not designed for a formal comparison. In adjusted analysis, having previously accessed one’s own medical record was associated with greater support for online access for all patients (odds ratio 4.74, 95% confidence interval 1.80–12.53). Age, gender, former professional setting and country showed no clear independent associations with support. Participants described benefits including patient empowerment, transparency, error correction and greater efficiency, alongside concerns about misinterpretation, anxiety and handling of sensitive information.

**Conclusions:**

In this cross-sectional convenience sample, retired doctors were more supportive of online access to health records than practising English GPs have reported in previous surveys. This cross-study difference cannot establish whether attitudes differ because of retirement, patient experience, professional role, sampling or other contextual factors. However, the association between having accessed one’s own record and support for access is consistent with the hypothesis that experience from the patient perspective may influence professional attitudes. Prospective comparative research is needed to examine this temporal relationship and to assess how record presentation and explanatory support affect patient and clinician experiences.

**Supplementary Information:**

The online version contains supplementary material available at https://doi.org/10.1186/s12911-026-03756-y.

## Introduction

Evidence indicates that providing patients with routine online access to their medical records—often termed online record access (ORA)—can support patient engagement, understanding, and involvement in care [1, 2]. A systematic review of access to records in acute settings concluded that verbal communication alone is frequently insufficient, and that access to written clinical information can complement consultations and support patient-centred care [3]. At the same time, concerns have been raised that access without explanation or context may increase anxiety, particularly when records contain provisional diagnoses, complex terminology, or unexplained test results [3].

Over the past decade, patient access to electronic health records has expanded internationally. Comparative analyses demonstrate substantial variation in policy, system design, and scope of access across countries [4]. It is now estimated that patients in at least 30 countries have some degree of online access to their health records [5]. The Nordic countries and the United States are among the most advanced, with high levels of routine use by patients [6, 7]. Studies from these settings suggest a recurring pattern: strong clinician scepticism prior to implementation, followed by more favourable views once access becomes established. For example, in the United States, clinician support for open notes increased markedly following implementation [8], while Swedish clinicians reported that many anticipated harms did not materialise in practice [9].

Despite this accumulating evidence, professional resistance remains prominent in some settings. In England, since October 2023 all general practices have been contractually required to provide adult patients with prospective, by-default access to their primary care records [2]. Nevertheless, implementation has been uneven, and professional bodies have expressed ongoing concerns about patient misunderstanding, safeguarding, and workload [10]. In a recent survey of practising general practitioners in England, only one-third regarded ORA as a good idea, with most anticipating increased anxiety among patients and additional demands on clinicians [11–13]. These concerns echo those reported in other countries prior to implementation.

Alongside primary care developments, access to hospital records remains fragmented in many health systems. In the UK, hospital data are often siloed across providers, and routine patient access has historically been limited. Earlier work showed that hospital doctors frequently overestimated the potential harms of patient access [14], while patients who accessed their records often found them helpful and reassuring. More recently, studies from Norway and elsewhere indicate substantial patient use of hospital portals where access is available [15]. In Spain, which has a similar health service to the UK, several regions have implemented patient portals providing access to hospital reports, imaging, and test results [16, 17], creating a contrasting context in which national policy is less centralised but practical access to hospital data may be more developed.

Beyond national policy differences, a more fundamental question remains underexplored: how do doctors themselves experience online record access when they are patients? Existing evidence is largely indirect. A review of doctors’ experiences of illness suggests that role reversal can reshape clinicians’ perspectives, challenging assumptions about information needs, uncertainty, and shared decision-making [18]. Retired doctors occupy a unique position in this regard. They are clinically literate and familiar with medical systems, but as older people more likely to be patients and so more likely to share the views of non-medically qualified patients.

Anecdotal accounts from retired clinicians suggested that attitudes toward ORA might differ between retired doctors who had and had not accessed their own records, and that experiences of access, particularly to hospital records, might differ between the UK and Spain. No previous study has examined retired doctors’ views on record access. To explore these issues, we conducted a mixed-methods survey of retired doctors in both countries, examining their experiences of accessing their own health records and their views on whether online access is appropriate for all patients.

### Aims

This study aimed to examine how retired doctors experience access to their own medical records as patients in the UK and Spain, and how these experiences relate to their views on whether online access is appropriate for all patients. By comparing two contrasting health system contexts and including doctors from primary and secondary care, we sought to better understand whether retired doctors had similar views to those previously reported for practicing UK GPs.

## Methods

The study was carried out in accordance with the Declaration of Helsinki. Ethical approval was obtained from the University of Plymouth Faculty of Health Ethics Committee (22/11/2024; Project 5786) and the Colegio de Médicos de Segovia (18/12/2024; Código CEI 24042). All participants gave informed consent.

Recruitment: This exploratory study used convenience and snowball sampling to recruit retired doctors in the UK and Spain. The aim was to compare patterns of experience across contexts rather than to generate population-representative estimates. Survey invitations were distributed through professional contacts and retired doctors’ networks in both countries. In the UK 16 medical societies said they would forward the invitation to members. In Spain, the Colegio de Médicos de Segovia circulated the invitation to its retired members. No financial incentives were offered.

Eligibility and consent: Criteria were prior qualification as a medical doctor, age ≥ 60 years, retirement from clinical practice, at least one contact with healthcare services in the UK or Spain in the previous year, and consent to the use of anonymous survey data. Eligibility and informed consent were confirmed through initial screening questions (Appendices 1 and 2).

Questionnaire and data collection: The anonymous online questionnaire included items on participants’ experiences of accessing their own medical records, their views on whether online record access is appropriate for all patients, and basic demographic characteristics. One attitudinal item was replicated from previous surveys of practising UK general practitioners to allow comparison [11]. The survey was administered using JISC Online Surveys [19] and was open from 11 January to 27 March 2025. There were 92 respondents from UK and 108 from Spain (Appendix 3).

Translation & reconciliation: The questionnaire was developed in English and translated into Spanish by members of the research team, with back-translation and reconciliation to ensure conceptual equivalence. Spanish-language free-text responses were initially translated into English using AI-assisted online translation tools. Translations were subsequently checked against the original Spanish responses and, where necessary, corrected by MSL, a bilingual clinician who has practised medicine in Spain and the UK and has professional experience as an English–Spanish medical translator. The reviewed English translations were used in the qualitative analysis.

Data quality checks: Although duplicate responses were considered unlikely, given the professional target population, responses were screened manually for unusually rapid completion times and identical free-text entries [20]. No responses were excluded following this review.

Quantitative analysis: Descriptive statistics and cross-tabulations were used to summarise experiences and attitudes. Logistic regression analyses explored factors associated with (i) having accessed one’s own medical records and (ii) support for online record access for all patients. Predictor variables included country, age group, gender, former professional setting (primary, secondary, other), recent health service use, and personal experience of record access. Full model specifications are provided in Appendix 8.

Qualitative analysis: Free-text responses were analysed thematically using an inductive approach (Appendices 5 and 6). Codes were developed iteratively and grouped into broader themes through repeated review. To facilitate integration with quantitative findings, themes were subsequently quantified to allow exploration of their relationship with overall support for record access [21]. Full coding frameworks and illustrative quotations are provided in the appendices.

Supplementary machine learning analysis: As a consistency check on the primary thematic analysis, an exploratory machine learning–based topic modelling analysis was conducted on the same free-text responses. This analysis was used to assess thematic convergence rather than as a primary analytic approach. Methods and results are reported in Appendix 7.

## Results

### Experience of accessing personal medical records

Overall, 98 of the 200 retired doctors (49%) reported having accessed their own medical records in the previous year (Table 1). Access was significantly more common among UK respondents than those in Spain (63% vs. 37%, *p* < 0.001) but there was no difference in the number of health service contacts (Appendix 4). In multivariable analysis (Appendix 8), country was the only independent predictor of having accessed records, with Spanish respondents substantially less likely to report access (Odds Ratio 0.34, 95% Confidence Interval 0.18–0.64). Age, gender, former professional setting (primary, secondary, other), and recent health service use were not significantly associated with access.

**Table 1:** Characteristics of retired doctors participating in the survey (*N* = 200)

Characteristic	UK (*n* = 92)	Spain (*n* = 108)	Total (*N* = 200)
**Age group, ** ***n*** ** (%)**			
60–69 years	34 (37)	55 (51)	89 (45)
70–79 years	45 (49)	38 (35)	83 (42)
≥ 80 years	13 (14)	15 (14)	28 (14)
**Female**,** n (%)**	31 (34)	52 (48)	83 (42)
**Former professional setting**,** n (%)**			
Primary care	41 (45)	34 (31)	75 (38)
Secondary care	43 (47)	63 (58)	106 (53)
Other / mixed	8 (9)	11 (10)	19 (10)
**Accessed own medical record in past year**, **n (%)**	58 (63)	40 (37)	98 (49)
**Support online access to records for all patients**,** n (%)**	63 (69)	65 (60)	128 (64)

*Notes*: Percentages may not total 100 due to rounding. Former professional setting refers to the main area of clinical practice prior to retirement. Statistical comparisons are reported in the Results and Appendices. We did not ask for further information on ‘other’ professional settings but these are likely to include public health, administration, and research among others

Qualitative responses highlighted differences in how records were accessed rather than in perceived value. UK respondents most frequently described routine digital access to primary care records via patient portals or apps, often supplemented by copied correspondence. Spanish respondents more frequently reported access to hospital records, including test results and reports, sometimes facilitated through informal professional contacts with colleagues still in practice. Despite these differences in access routes, respondents in both countries described broadly similar experiences of access.

Among those who had accessed their records, the vast majority (86/98) found this useful. Reported benefits included greater autonomy and control, reassurance and peace of mind, the ability to prepare for consultations, avoidance of unnecessary contact with services, and identification of errors or omissions. A small minority expressed dissatisfaction, mainly related to incomplete, fragmented, or poor-quality records. Most respondents felt that access was more useful to them as retired doctors because of their clinical understanding, although several emphasised that access should be available equally to all patients regardless of background.

### Views on online access to records for all patients

Overall, 64% of retired doctors supported online access to medical records for all patients. Support was slightly higher among UK respondents than Spanish respondents (69% vs. 60%), but this difference did not reach statistical significance. Levels of support were substantially higher than those reported previously among practising UK general practitioners.

Qualitative responses revealed consistent patterns across both countries. Respondents who supported access emphasised patient empowerment, autonomy, and informed participation in care. Many framed access as a matter of patient rights, transparency, and trust in public institutions, while others highlighted practical benefits such as improved understanding, shared decision-making, error correction, and reduced need for follow-up contacts. UK respondents more frequently emphasised transparency, trust, and error correction, whereas Spanish respondents more often framed access in terms of patient rights and ownership of data.

Among respondents who opposed universal access, the dominant concern was the risk of misinterpretation of medical information by patients without sufficient clinical knowledge. Some expressed concern that access could increase anxiety, particularly when viewing abnormal results or sensitive information without explanation. Relatively few respondents raised concerns about additional workload for clinicians. Several respondents supported access in principle but argued that safeguards, contextual explanation, or exceptions for specific patient groups might be necessary.

### Factors associated with online record access

In multivariable logistic regression analysis (Appendix 8), prior self-reported access to one’s own record showed the largest adjusted association with support for online access for all patients (Odds Ratio 4.74, 95% Confidence Interval 1.80–12.53). Participants in this sample who had accessed their own records were nearly five times more likely to support wider access than those who had not.

Other factors, including country, age, gender, former professional setting (primary, secondary, other), and recent health service use, were not significant independent factors associated with support. A borderline trend suggested greater support with increasing age, but this did not reach conventional levels of statistical significance. There was no significant interaction between country and personal record access, indicating that the association between self-access and support was consistent across both the UK and Spain.

Although UK respondents were more likely than Spanish respondents to have accessed their own records, overall support for access did not differ significantly by country once personal experience was considered. Within this model, prior self-reported access had the largest adjusted association with support but the cross-sectional data cannot establish whether access influenced attitudes, whether pre-existing attitudes influenced access, or whether both reflect other factors.

## Discussion

This study is the first to examine retired doctors’ views on online patient access to medical records in two similar but contrasting European health systems. We found that our sample of retired doctors in both the UK and Spain were substantially more supportive of online record access than practising UK general practitioners reported in recent surveys [11–13]. Whereas only around one third of practising GPs in England have described online record access as a good idea [11], nearly two thirds of retired doctors in our study supported access for all patients. This was an exploratory study with an opportunistic sample, but the contrast is worthy of further investigation. For example, among practising doctors, is prior personal use of online record access associated with views about access for patients? How, if at all, do doctors’ views change across retirement, and are any changes associated with measured workload, medicolegal, organisational or other contextual factors?

Across the UK and Spanish respondent samples, prior self-reported access to one’s own record was associated with support for wider access after adjustment for the measured covariates. This association is compatible with the possibility that experience as a patient influences doctors’ attitudes, but the design cannot establish temporal order or causal direction. Doctors already favourable to ORA may have been more likely to seek access, and unmeasured factors may contribute to both.

Despite differences in health system organisation and digital infrastructure, overall support for record access was strikingly similar in the UK and Spain once personal experience was taken into account. UK respondents were more likely to have accessed their records digitally, typically through primary care portals, whereas Spanish respondents more often described access to hospital records, sometimes facilitated through informal professional networks. These differences in access routes reflect variations in policy maturity and system design but did not translate into substantial differences in attitudes toward access itself. This suggests that opportunity to engage with one’s own record may be more influential than the specific technical or organisation pathway through which access occurs.

Qualitative findings help explain this pattern. Across both countries, retired doctors who supported access emphasised patient empowerment, transparency, trust, and the practical benefits of being able to review results, correct errors, and prepare for consultations. These themes mirror those reported by patients in earlier studies [22]. Concerns among respondents who opposed universal access resembled those reported previously by primary-care clinicians and staff, including anticipated patient misunderstanding, anxiety arising from information viewed without clinical context, and challenges in managing sensitive, provisional or safeguarding-related information [12, 13].

Respondents frequently recognised that their clinical training made records easier for them to interpret. This did not necessarily determine their views on universal access: some regarded limited clinical literacy as a reason to oppose access for all patients, whereas others supported universal access despite acknowledging that they themselves derived additional benefit from their medical expertise. Clinical literacy should therefore be understood as shaping the perceived utility and risks of access, rather than as a straightforward explanation for support for universal access.

Studies from the Nordic countries and the USA show strong clinician scepticism prior to implementation of ORA, followed by more favourable views once access becomes established [6–9]. While practicing GPs in the UK expressed concern about workloads [12] relatively few of our retired respondents emphasised workload burden. This difference suggests that some resistance to record access may be linked to workload pressures and organisational constraints rather than to fundamental objections to patient access. Recent work has highlighted the potential role of digital tools, including generative approaches, in supporting patient understanding of clinical records, although evidence of effectiveness and safety remains limited [23].

Including retired doctors from both primary and secondary care backgrounds strengthens the interpretation of our findings. Former professional setting was not clearly associated with support in this sample. Retired doctors occupy a unique position: they are able to interpret medical information, yet no longer carry responsibility for documentation, medico-legal risk, or service delivery. Their experience as a patient may influence their attitude but this study cannot determine how their attitudes developed or changed over time.

These findings have implications for policy and professional education. As health systems move toward more open records, technical implementation alone is unlikely to resolve clinicians’ concerns. Although this study cannot show that experiential learning changes attitudes, the observed association identifies this as a possible intervention to test in prospective research. Greater emphasis may be needed on experiential learning and engagement, including opportunities for clinicians to reflect on record access from the patient perspective. Educational initiatives could focus on how to explain records clearly, anticipate patient questions, and use access as a tool to support communication rather than as an additional burden. At the same time, improving patient-facing explanations and contextual information may help address concerns about misunderstanding and anxiety.

Recent work has proposed the use of generative artificial intelligence, including large language models, to help patients interpret complex clinical records and reports; however, evidence of effectiveness, safety and acceptability in routine care remains limited [23, 24]. While such tools may help patients navigate complex information, they also raise questions about accuracy, accountability, and trust. Our findings suggest that technological solutions should complement, rather than replace, professional judgement and communication, and that clinician acceptance is likely to depend on how well these tools align with clinical workflows and patient needs.

This study has limitations. As a non-probability, online survey using convenience and snowball sampling, the findings are not population-representative and may over-represent digitally engaged retired doctors. Invitations were sent both to personal networks and to medical societies. Although we think most responses resulted from the latter as an anonymous survey we cannot be sure. Although our invitations to personal networks was ‘non-leading’ and responses were anonymous (so no reason for a colleague to respond ‘favourably’), some may argue that this over-represents those in favour of open access. Although Spanish-language responses were checked and corrected by a bilingual clinician with professional medical translation experience, some linguistic, idiomatic, or culturally specific nuance may have been attenuated through translation. The UK and Spanish samples differed demographically, limiting the strength of direct cross-national comparisons. All data were self-reported and cross-sectional, precluding causal inference. In particular, the study cannot determine whether self-access preceded support, whether support preceded self-access, or whether the association resulted from unmeasured confounding or selection effects. The findings should therefore be interpreted as exploratory associations among respondents.

In summary, retired doctors in both the UK and Spain appear more supportive of online patient access to medical records than practising clinicians, and this support is strongly associated with personal experience of access rather than with professional background or national context. As health systems continue to expand patient access to records, strategies that emphasise experience, education, and communication may be as important as technological capability in shaping professional acceptance.

## Conclusions

The retired doctors in our samples in the UK and Spain were much more supportive of online patient access to medical records than practising clinicians reported in recent studies. Across both countries, support was associated primarily with personal experience of accessing one’s own records rather than by former professional role or national context. Although access routes differed, being more digitally routine in the UK and more informal in Spain, these differences were not independently associated with attitudes once experience was taken into account. This association cannot establish whether experience of access influenced attitudes, whether pre-existing attitudes influenced access, or whether other factors explain both. Prospective comparative research is needed and exploration of strategies that use experiential learning should be considered.

## Supplementary Information

Below is the link to the electronic supplementary material.

Supplementary Material 1

## Data Availability

Anonymous, de-identified data will be made available on the University of Plymouth Centre for Health Technology website upon acceptance. Appendices include the full English and Spanish questionnaires.

## Declarations

### Public and patient involvement

Four of the research team (BF, RF, AL, MSL) were retired doctors involved in all aspects of the research from conception, through to dissemination.

## Abbreviations

UK: United Kingdom
ORA: Online Record Access
