# Supplementary material for: Retired doctors as users of patient-facing electronic health records: a mixed-methods survey in the UK and Spain
Source: BMC Med Inform Decis Mak. 2026 Aug 14;26:462. doi: 10.1186/s12911-026-03756-y (PMC13757020; doi:10.1186/s12911-026-03756-y)
Supplement: Supplementary file 1 [file 12911_2026_3756_MOESM1_ESM.docx]

# Retired doctors as users of patient-facing electronic health records: a mixed-methods survey in the UK and Spain

# Appendices 1-8

Ray Jones¹^*^, Angeles Lazcoz^2^, Shangming Zhou¹, Brian McMillan^3^, Olanrewaju Bamidele¹, Richard Fitton^2^, Brian Fisher^2^, Mar Soler-Lopez², Charlotte Blease^4^

¹ Centre for Health Technology, University of Plymouth, Plymouth, PL4 8AA, UK
² Retired doctor

^3^ Division of Population Health, Health Services Research & Primary Care, School of Health Sciences, University of Manchester, Manchester, M13 9NT, UK

^4^ Department of Women's and Children's Health, University of Uppsala, Akademiska sjukhuset 751 85 UPPSALA, Sweden

*Corresponding author: Ray Jones ([ray.jones@plymouth.ac.uk](mailto:ray.jones@plymouth.ac.uk))

Centre for Health Technology, School of Nursing and Midwifery, Plymouth PL4 8AA

**Appendix 1: Survey Questions – Retired Doctors' Views on Patient Access to Records**

| **Question** | **Question Text** | **Response Type** |
| --- | --- | --- |
| 1 | Did you qualify as a medical doctor? | Yes/No |
| 2 | Are you aged 60 or over? | Yes/No |
| 3 | Are you retired from clinical practice? | Yes/No |
| 4 | Have you had contact with any health care providers in the UK and/or Spain at least once in the last year? | Yes/No |
| 5 | Do you agree to your anonymous data being used in this study? | Yes/No |
| 6 | Have you seen ANY of your own medical records in the last year? | Yes/No |
| 7 | Please tell us more about what type of medical record you saw, where, when, and how you accessed (saw) them. | Open-ended |
| 8 | How long did it take to get access? Was it instantly available or did you have to 'apply for' access? | Open-ended |
| 9 | Did you have to pay to access your records? If so, roughly how much? | Open-ended |
| 10 | Do you feel it was easier for you as a retired doctor to help gain access? If so, please tell us more. | Open-ended |
| 11 | Did you find accessing your record useful? If yes, how? If not, why not? Which records were or were not useful to see? | Open-ended |
| 12 | Did you try to access some medical records without success? What happened? | Open-ended |
| 13 | Do you think being able to access your record is more useful or less useful to you as a retired doctor compared to other patients? Why? | Open-ended |
| 14 | Do you think online open access to their own medical record for all patients is a good idea? | Yes/No |
| 15 | Why do you think it is a good idea? | Open-ended |
| 16 | Why do you think it is not a good idea? | Open-ended |
| 17 | What is your age group? | Multiple choice: Under 60 / 60–69 / 70–79 / 80 or over |
| 18 | What is your gender? | Multiple choice: Male / Female / Other or prefer not to say |
| 19 | What was the location of your last clinical practice post as a doctor? | Multiple choice: General Practice / Hospital / Other |
| 20 | Approximately how many contacts with health services did you have as a patient in the last year? | Multiple choice: 3 or fewer / 4–10 / 11 or more |
| 21 | In which country did you have contacts as a patient in the last year? | Multiple choice: UK / Spain / Both UK and Spain |
| 22 | In which UK regions and/or Spanish autonomies were your contacts as a patient? | Open-ended |
|  |  |  |

**Appendix 2 Survey Questions: Spanish version**

| Pregunta | Texto de la Pregunta | Tipo de Respuesta |
| --- | --- | --- |
| 1 | ¿Calificaste como médico? | Sí / No |
| 2 | ¿Tiene usted 60 años o más? | Sí / No |
| 3 | ¿Está usted retirado de la práctica clínica? | Sí / No |
| 4 | ¿Ha tenido contacto con algún proveedor de atención sanitaria en el Reino Unido y/o España al menos una vez durante el último año? | Sí / No |
| 5 | ¿Está de acuerdo con que sus datos anónimos sean utilizados en este estudio? | Sí / No |
| 6 | ¿Ha visto ALGUNA de sus propias historias clínicas durante el último año? | Sí / No |
| 7 | Cuéntenos más sobre qué tipo de historias clínicas vio, dónde, cuándo y cómo accedió a ellos. | Abierta |
| 8 | ¿Cuánto tiempo tardó en conseguir el acceso? ¿Estuvo disponible instantáneamente o tuvo que "solicitar" acceso? | Abierta |
| 9 | ¿Tuvo que pagar para acceder a su historia clínica? Si es así, ¿cuánto aproximadamente? | Abierta |
| 10 | ¿Cree que fue más fácil para usted, como médico jubilado, ayudar a obtener acceso? Si es así, cuéntenos más. | Abierta |
| 11 | ¿Le resultó útil acceder a sus historias clínicas? En caso afirmativo, ¿cómo? Si no, ¿por qué no? ¿Qué registros fueron o no útiles? | Abierta |
| 12 | ¿Intentó acceder a algunas historias clínicas sin éxito? ¿Qué pasó? | Abierta |
| 13 | ¿Cree que poder acceder a su historia clínica le resulta más o menos útil como médico jubilado en comparación con otros pacientes? ¿Por qué? | Abierta |
| 14 | ¿Cree que el acceso abierto online a su propia historia clínica para todos los pacientes es una buena idea? | Sí / No |
| 15 | ¿Por qué cree que es una buena idea? | Abierta |
| 16 | ¿Por qué cree que no es una buena idea? | Abierta |
| 17 | ¿Cuál es su grupo de edad? | Menos de 60 / 60–69 / 70–79 / 80 o más |
| 18 | ¿Cuál es su género? | Masculino / Femenino / Otro o prefiero no decirlo |
| 19 | ¿Cuál fue la ubicación de su último puesto de práctica clínica como médico? | Medicina General / Hospital / Otro |
| 20 | ¿Cuántos contactos con servicios de salud tuvo como paciente en el último año? | 3 o menos / 4–10 / 11 o más |
| 21 | ¿En qué país tuvo contactos como paciente en el último año? | Reino Unido / España / Ambos |
| 22 | ¿En qué regiones del Reino Unido y/o autonomías españolas fueron sus contactos como paciente? | Abierta |

**Appendix 3. Location of respondents**

| **UK** | | **Spain** | |
| --- | --- | --- | --- |
| Scotland | 29 | Rural Central Spain | 67 |
| Southwest England | 24 | Madrid | 32 |
| NE & NW England | 11 |  |  |
| London, SE England, S coast England | 10 |  |  |
| N Ireland | 12 |  |  |
| Wales | 2 |  |  |
| Other England | 2 | Other Spain | 6 |
| Missing | 2 | Missing | 3 |
| **TOTAL** | **92** | **TOTAL** | **108** |

**Appendix 4 Number of contacts by age group and country**

| (Q20) Number of health service contacts | | Age group (Q17) | | | Total |
| --- | --- | --- | --- | --- | --- |
|  |  | 60-69 | 70-79 | 80+ |  |
| UK | 3 or fewer | 15 | 18 | 5 | 38 |
|  | 4-10 | 17 | 21 | 10 | 48 |
|  | 11 or more | 2 | 4 | 0 | 6 |
| Spain | 3 or fewer | 27 | 18 | 1 | 46 |
|  | 4-10 | 26 | 24 | 2 | 52 |
|  | 11 or more | 5 | 5 | 0 | 10 |
| Total | 3 or fewer | 42 | 36 | 7 | 84 |
|  | 4-10 | 43 | 45 | 12 | 100 |
|  | 11 or more | 7 | 9 | 0 | 16 |
| Total | | 92 | 90 | 18 | 200 |

No significant association. Chi-square=2.5; 4df; p=.638

**Appendix 5 Coding schemes and quantification of thematic analysis for open questions on respondents own experience of accessing records (Q7-Q13)**

Left hand column shows, code letter (as appears on available datafile), number of respondents with this theme (comments made were given multiple themes), and if Spain or UK had this type of comment made more often (based on chi squared from crosstab site Vs presence of comment). +++ p<0.001, ++ p<0.01, + p<0.05

**Q7 Please tell us more about what type of medical record you saw, where, when, and how you accessed (saw) them**

All 98 people who had said they had seen their records provided some comment but comments from UK respondents contributed more themes (mean 3.4) than Spanish respondents (mean 2.5) (t=3.0; 96df; p=0.002).

| **Code**  **Count** | **Theme** | **Examples** |
| --- | --- | --- |
| A  57  UK  +++ | **Location: GP/Primary Care/Health Centre** | - Able to access full GP medical record via airmid app from GP surgery (xxxx Medical Centre). - GP records from childhood. Photocopies. Fascinating! And useful for current problems here in xxxxx. |
| B  49  Spain  + | **Location: Secondary care** | - Data from my history at the General Hospital of xxx provided by other colleagues - Hospital reports and test results are accessible in the Patient Folder of the xxxx Health Service. I can also see a summary of my primary care history and my vaccination record. |
| C  44  UK  ++ | **Routine access: via app, portal or copied letters**: NHS App, Patient Access, Airmid, Sacyl Conecta, Mi Carpeta de Salud, Tarjeta Sanitaria Virtual | - Fundación XXX at any time through the App - GP health record on the NHS app - Hospital letters I am copied into and health records on NHS app and MyChart, the app associated with the hospital EPIC IT system - Like most patients, I am sent a copy of the GP letter from consultants to my GP |
| D  12 | **Requested access: personal** via clinician during appointment, by colleague, or after via WhatsApp or email | - My family doctor at the xxx Health Center, who has been my colleague for years, showed me in my medical history the results of my latest tests, intraocular pressure, as well as my pending ophthalmology appointments. In short, everything I had requested - With my former colleague, my family doctor at the XXXX Health Center. At the General Hospital of XXX I request and receive reports instantly after consultations |
| E  31  UK  +++ | **Basic data accessed:** GP records, blood test results, prescriptions, vaccination history, | - Access to blood test results and consultation records and clinic letters from my GP in a village near XXX via the NHS app - gp record, clinic letters, results, prescriptions, vaccinations all via nhs app |
| F  42  UK  +++ | **More detailed data accessed**: letters, reports, summaries: hospital letters, discharge summaries, primary care summaries | - I asked for copies of the pathology report on my operative specimens and my scans reports from the xxx Hospital - I saw an MRI scan. A letter from a consultant who reviewed me and blood results from a GP. |
| G  39 | **Advanced data accessed:** advanced: imaging reports, lab and imaging results, specialist reports, full clinical documentation | - Complementary tests, lab tests, CT scans, mammograms, general surgeon in Madrid - Consultant letters and xrays - I accessed laboratory results, complementary tests, and anatomical pathology results through contacts at the hospital in my city |
| I  18  Spain  + | **Use cases**: Self-management of conditions, understanding, medication adjustments, reviewing historical context, second opinions, correcting errors, travel insurance, curiosity | - Hematology service medical history at the Hospital of xxx and University Hospital of xxx. I accessed it by requesting it from the corresponding hematologist available at the time - I needed access to my records in July to fill in a travel insurance application and answer specific questions. I also saw a specialist privately in Bristol and needed to take my latest blood results when I saw him. |
| J  3 | **Poor Quality of records**: Data fragmented, missing, incorrect, inaccessible or incomprehensible | - 1. 2ry care [private] orthopaedic secretary XXX sent me a copy of his letter to my own gp in XXXh, after I asked. Not automatic. 2. After 3 requests I was sent a secure link to my last years gp medical record broken down into 10 sections and in p10 separate files. So a fragmented record - I requested that a specialist at the General Hospital correct some incorrect data. |

**Q8 coding: How long did it take to get access? Was it instantly available or did you have to 'apply for' access?**

| **Code**  **Count** | **Theme** | **Examples** |
| --- | --- | --- |
| A  61 | **Quick:** Instant, efficient, and automatic access once setup was complete | - Spontaneous - Instantly - Applied for access and they were made readily available - Entering my data and key, it took little time |
| B  17 | **Set up challenges:** having to apply in person, provide photo ID, complete paper forms, process frustrating, multiple visits or calls, need for digital certificates, access codes, or in-person visits to activate access. | - This was a couple of years ago but it took about 4 phone calls and 3 visits to the surgery to set it up! - I had to apply in person with ID at my surgery - It took me a while to access it because they asked for an access key |
| C  12  UK  + | **Copy letters:** Receiving test results or letters directly from GPs or consultants | - Consultant letters usually arrive unbidden within a month. Sometimes I have had to request them from the Secretary - In the form of a copy letter of the original sent to the GP |
| D  10  Spain  ++ | **Via someone:** Accessing records during or after consultations, or with help from colleagues. | - At the moment, through patient care staff when I moved my residence to another community - Alongside a former colleague still active - Had to request access from colleagues |
| E  5 | **Usability**: Not user-friendly (eg poor structure, things in the wrong place, or need to search manually through the system using keys or certificates), only partial access (e.g., only seeing results, not full notes) | - The software is not very good, and it was a bit difficult for me to access my medical history - The online "My Care" system routinely gives access to routine tests, FBC, U&E, etc., but it is quirky and unreliable. Hospital test results appear quickly, but General practitioner-requested tests may take weeks. - I had access to my clinical reports but not to the full medical records themselves |
| F  6 | **Other**: unclear |  |

**Q10 Do you feel it was easier for you as a retired doctor to help gain access? If so, please tell us more.**

| **Code**  **Count** | **Theme** | **Examples** |
| --- | --- | --- |
| B  40 | **No.** Either as simple answer or embedded in longer answer perhaps mentioning equality of access. Statement that any patient can access their records, suggesting universal access is expected, so retired doctors are no different | - No - All patients with the practice can access their own records. Being a retired doctor makes no difference. - In public healthcare, it is the same for everyone - Doesn't make any difference who you are, and I don't think it should |
| C  19 | **Yes** – simple statement that it was possible or likely that being a retired doctor helped gain access without much other justification | - Yes - Probably easier although when I was a consultant I always copied all letters to the patient from 1976 onwards - sometimes it was easier but not always - Yes I think it was easier as a retired doctor to ask for and receive a copy letter |
| D  17  Spain  +++ | **Professional Advantage via individuals**. Relying on former colleagues, former workplace ties, or being known in the system to gain easier or quicker access. Trusting the system and practising colleagues so removing the need to access records personally | - I can request it from a colleague - I think it was easier for me because it was a request among colleagues in the profession - It was easier for me as a retired doctor to obtain access with the help of acquaintances or previous connections - Yes, it was more accessible by requesting the favor from colleagues - Yes, although retired, I still have many contacts. My general practitioner was one of my medical school tutees. |
| E  9 | **Knowledge of the System.** Their familiarity meant they had a better understanding of the system worked, where to look, whom to contact | - As a retired GP I understood the system for gaining access and knew what was possible - I knew I had a right to them, and I knew to ask, and keep asking… and i will now challenge them |
| F  6 | **Perception of retired doctor**. Working colleagues assuming that retired doctor would want access, or more details, could better interpret results | - I was known to be a doctor and I think access was easier but I have no proof of this |
| G  14 | **Other or unclear**. | - I trust my hospital consultants and feel it is their job to keep my records up-to-date and accurate, not mine! |

**Q11: “Did you find your record useful to access? If yes, how, if not, why not? Which records were or were not useful to see?”**

Only 5/94 who commented, dd not find something useful. There was no difference between Spain and UK on the three main categories.

| **Code**  **Count** | **Theme** | **Examples** |
| --- | --- | --- |
| A  81 | **Usefulness and satisfaction** where particular reason distinguished shown below C-G | - Yes - Yes, because I was looking for some data |
| H  8 | **Yes but.....**satisfied but pointing out problems of quality | - What I could access was very useful, but it was incomplete. Some test results were missing - Blood test results - very useful and helpful. Letters, operation notes, discharge summaries - sometimes helpful to confirm dates of procedures, etc., but otherwise not interested. I'm also surprised about that when inaccuracies get listed electronically (e.g. details of a procedure) it is practically impossible to rectify the record, however many times one points it out! . |
| B  5 | **Dissatisfaction** - not useful, mostly because of poor quality data | - It wasn't useful at all because it was old, incomplete, and useless data for what I wanted - No, because it was incomplete and certain tests like mammograms were supposed to be sent to me by mail but never arrived - My old ones. The online ones are very difficult. |
| **Sub categories of usefulness** | | |
| C  12 | **Useful allowing more autonomy** and decisions to be made | - Yes, useful for pathology results as no follow-up contact with the practice required - Yes - I was able to make the clinical decision not to pursue further tests and was reassured - A written record… allows me to feel in control |
| D  4 | **Useful for peace of mind** | - It was useful for my medical peace of mind - It helped ease anxiety about results and clinical problems - It was useful because it helped ease anxiety about results and clinical problems |
| E  1 | **Useful allowing preparation for appointments** | - I could see blood test results, imaging test reports, and pathology reports before having a medical appointment |
| F  4 | **Useful enabled avoidance of having to contact practice** | - Able to access pathology results without ringing to ask - Yes, I could see the test results before going to the doctor - Very useful. I can check what has been added… and order my repeat medications |
| G  12 | **Useful as reminder of the consultation** | - It was useful in some cases to realize clinical information I hadn’t picked up during consultations, and also in some cases to confirm doubts about the treatment to follow - It was useful to see the reports directly, in addition to knowing the result from my doctor’s verbal information |

**Q12 “Did you try to access some medical records without success? What happened?”**

| **Code**  **Count** | **Theme** | **Examples** |
| --- | --- | --- |
| A  116  UK  +++ | **No problem** | - No. Was able to access my wife's records, with her agreement - No - Always successful |
| B  29  Spain  + | **Had not attempted to access records** | - Honestly, I haven’t tried - I didn't try |
| C  9  Spain  ++ | **Had not attempted to access records because of low expectations of success** | - I don't have access - No possible access They’re not accessible |
| D  13 | **Hospital / specialist reports not available** | - Reports from specialists are not uploaded to the system - Yes, hospital results, not available - Nurse at clinic said she was unable… I need to ask consultant/secretary - I couldn’t access my ophthalmology history because it’s not digitized - Reports from specialists are not uploaded to the system, and you don’t have access to them - I would like to be able to see the results of tests that I have had done at the hospital. These are not directly available to patients |
| E  12 | **Technical barriers** | - The health app doesn’t give me access… sometimes due to app issues - Although App did not work initially I asked for paper copies - The health app doesn’t give me access to the information. Sometimes due to app issues |
| F  4 | **Missing, incomplete, or incorrect data** | - Sometimes because the electronic record is incomplete |
| G  4 | **Easier route via informal solutions (colleagues or staff)**  Spain  + | - You always have to ask a colleague who is still working - No. It’s not easy to access, and the information provided by the doctor was sufficient - I can ask my family doctor, who gives me verbal information |
| H  8 | **Barriers undefined** | - Yes, ignored… I had to persist - I couldn’t access it |
| J  14 | **Other** | - I tried to access some records through the main hospital system during a pilot experiment more than 10-15y ago. It was clunky, incomplete, lacking contextual information |

**Q13 “Do you think being able to access your record is more useful or less useful to you as a retired doctor compared to other patients? Why?”**

No differences in category counts between UK and Spain

| A  140 | **More useful** due to clinical understanding and ability to act upon it | - More useful because I can interpret test results - It’s more useful because I can interpret test results, which most patients can’t - Yes. I understand reports better - Yes, because I can evaluate other diagnostic, treatment, and prognosis options - Yes. To better understand my health, engage more constructively with my clinicians and to correct any errors - Yes, more useful. I could spot and correct possible errors |
| --- | --- | --- |
| B  7 | Regardless of being more useful all patients should have **equal access** | - I don’t think I should access my records differently from how my patients do - No, all patients should have access |
| C  8 | **Equally useful** | - No difference between doctor or non-qualified patient |
| D  39 | **Not very useful** | - It’s very complicated to access one’s own records |
| E  1 | **Caution about interpretation** of own medical record as a doctor | - As a patient, I am aware that I cannot be totally objective about my own health... I respect and rely on the opinions of other professionals |
| F  11 | **Other** comments more related to other questions | - Access can allay worries or identify legitimate issues with care |

**Appendix 6 Thematic coding of views on whether ORA is a good idea for all patients (Q15, Q16)**

**Q15 Why do you think it is a good idea?**

| A  31  Spain  ++ | **Rights:** Patient rights and ownership, human rights, freedom of information, and transparency in public institutions. | - The medical history does not belong to the doctor, it belongs exclusively to the patient - It is the patient’s own data so it’s a human rights issue |
| --- | --- | --- |
| B  52 | **Empowerment and autonomy:** access promotes self-awareness, shared responsibility, informed participation in care, confidence, education, and health ownership | - Helps with awareness and motivates patients to take ownership of their health - Empowers patients... improves standards of care... shared responsibility for health |
| C  17  UK  +++ | **Transparency and Trust:** patients may feel deceived if records are hidden; openness is essential to doctor–patient trust, reduces complaints and errors | - Because it would reflect a more believable reality—sometimes patients may feel deceived - Transparency and openness should be the default... improves what doctors write about the patient |
| D  18 | **Practical Benefits** including avoiding test duplication, facilitating second opinions, GP workload, saving time, identifying errors, better communication across services. | - They can see test results... avoid repeating blood tests or imaging - Takes pressure off GPs... reduces time spent calling about results |
| E  26 | **Informed Decision-Making**: Encourages better understanding and treatment adherence, necessary for informed discussions, shared decision-making, and accurate understanding of one's health condition | - Improves knowledge, encourages shared responsibility, self-care, and adherence to treatment - It is essential for informed discussion and decision-making |
| F  11  UK  ++ | **Error Correction and Accuracy**: access allows patients to challenge inaccuracies, clarify misunderstandings, and improve safety | - Allows them to ask their doctor more thoughtful questions - We can challenge inaccurate and incomplete records |
| G  8 | Supporting access but with **limitations and safeguards:** sensitive or subjective content - not all data should be visible without context; third-party data, and information overload for patients not ready for clinical detail | - There shouldn’t be access to subjective notes... - There may be occasions when some information needs to be held back... psychiatric consultations are an obvious example |

**Q16 Why do you think it is not a good idea?**

| A  43  Spain  + | **Risk of Misinterpretation and Lack of Medical Knowledge**; patients don’t understand medical language, which could lead to confusion | - Too much medical information causes misinformation |
| --- | --- | --- |
| B  13 | **Potential Harm to Patient Wellbeing**: concerns focus on increased anxiety, emotional distress, and misunderstood test results, especially if accessed without medical guidance, serious diagnoses, sensitive notes, or mental health content | - Full access to all data may cause more anxiety than benefit - You might have a terrible result… better explained sympathetically by a doctor |
| C  3 | **Impact on how doctor writes history**; patient access might influence how clinicians write or cause misunderstanding of subjective notes, would inhibit frank documentation or change the tone of medical records to avoid patient offence | - The notes… would be influenced by concerns about potential misinterpretation - Doctors would be inhibited… patient had access |
| D  2 | **Additional Workload for Clinicians;** extra explanations might burden professionals GPs would face more queries, need to reword records, or spend time explaining benign findings | - Patients would seek explanations… leading to a significant workload for doctors - It will create a lot of extra work for GPs… |
| E  3 | **Privacy and Data Security**; concern about third-party visibility or manipulation,  cybercrime, coercion (e.g. in domestic abuse), and unauthorised sharing | - What is online is more vulnerable to being seen by third parties - Concerned about cyber crime and hacked records |
| F  5 | **Patients need guidance and explanation**: viewing records with a doctor would aid understanding, access should be accompanied by explanation, or restricted for certain groups (e.g. people with mental illness) | - Ideally, it should first be reviewed with a doctor who can interpret it - Access… better done with medically qualified staff available for explanation |
| G  6  UK  + | **Exceptions based on patient characteristics**; differentiation by patient type, especially those with psychiatric or cognitive vulnerabilities | - There might be groups of patients… for which it might not be a good idea |
| H  4 | **Mixed or Uncertain Views** | - I’m honestly not sure whether it’s a good or bad idea - I have mixed feelings about this…” |

**Appendix 7 (BERTopic analysis)**

BERTopic is a modern, machine learning–based approach for discovering hidden (“latent”) themes in large sets of open-ended text responses. It works by representing each response as a numerical vector that captures its meaning; this is done using transformer-based sentence embeddings (for example, the MiniLM models). BERTopic then groups together responses with similar meanings using clustering algorithms (typically HDBSCAN). Finally, it identifies the most characteristic keywords for each cluster, which helps interpret and label the emerging themes. BERTopic combines recent advances in natural language processing with topic modelling making it well suited for analysing qualitative survey data, as it can handle nuanced language, short answers, and overlapping themes. Q15 (positive experiences) responses were modelled using default BERTopic settings with all-MiniLM-L6-v2 sentence-transformer embeddings. Outliers (topic = -1) were excluded, resulting in five coherent themes. Q16 (negative experiences) topic modelling was run twice: first using the same all-MiniLM-L6-v2 embeddings for comparability, and then with paraphrase-MiniLM-L6-v2 embeddings to test robustness. After adjusting the min_topic_size parameter, four meaningful themes were identified. Reference: Grootendorst, M., BERTopic: Neural topic modeling with a class-based TF-IDF procedure. arXiv, 2022. preprint arXiv:2203.05794. BERTopic: Neural topic modeling with a class-based TF-IDF procedure.

**Five themes supporting ORA from BERTopic analysis**

**History & Data Access** Patients believe access to their medical history improves responsibility & care

**Timeliness & Health Benefit** Access helps save time, allows error checking, improves patient health literacy

**Transparency & Records** Advocates for transparency and equal rights to view personal medical records

**Information Rights** Highlights the right to health information and its role in awareness

**Being Better Informed** Access leads to better understanding and more informed decision-making

**Four themes showing concerns about ORA from BERTopic analysis**

**Doctor Mediation & Data complexity** Concerns that patients may misinterpret complex medical data without guidance from healthcare professionals

**Misinterpretation & Knowledge gaps** Highlights the risk of confusion or misinterpretation due to limited medical literacy

**Anxiety & Emotional Risk**

Suggests that open access could trigger anxiety or emotional distress, especially in vulnerable groups

**Confidentiality & Ethical concerns** Raises ethical and privacy concerns about exposing sensitive or third-party health data

**Appendix 8. Logistic Regression Results**

**Self-access of online medical records**

Table A8.1. Multiple logistic regression predicting self-access of online medical records among retired doctors in the UK and Spain (N = 199). (One respondent did not answer Q14). Odds ratios (OR) > 1 indicate greater likelihood of having accessed own records. The model was statistically significant (χ²(8) = 20.6, p = 0.008), Hosmer–Lemeshow p = 0.62, Nagelkerke R² = 0.13.

| **Predictor** | **OR** | **95% CI** | **p** |
| --- | --- | --- | --- |
| Country (Spain vs UK) | 0.34 | 0.18 – 0.64 | <0.001 |
| Age group (70–79 vs 60–69) | 0.64 | 0.33 – 1.23 | 0.187 |
| Age group (80+ vs 60–69) | 0.72 | 0.22 – 2.36 | 0.586 |
| Gender (Female vs Male) | 0.75 | 0.39 – 1.45 | 0.402 |
| Professional background (Secondary vs Primary) | 1.36 | 0.70 – 2.65 | 0.367 |
| Professional background (Other vs Primary) | 1.25 | 0.41 – 3.82 | 0.692 |
| Health service contacts (4–10 vs ≤3) | 1.56 | 0.84 – 2.90 | 0.158 |
| Health service contacts (≥11 vs ≤3) | 2.56 | 0.80 – 8.19 | 0.113 |
| Constant | 1.54 | – | 0.259 |

Note. OR = odds ratio; CI = confidence interval. Reference categories: UK (country), age 60–69 (age group), Male (gender), Primary care (professional background), ≤3 service contacts (health service contacts).

**Support for online record access**

# Table A8.2. Multiple logistic regression predicting support for online record access (ORA) among retired doctors in the UK and Spain (N = 199). Odds ratios (OR) > 1 indicate greater likelihood of supporting ORA. The model was statistically significant (χ²(7) = 24.7, p < 0.001), Hosmer–Lemeshow p = 0.57, Nagelkerke R² = 0.16.

| Predictor | OR | 95% CI | p |
| --- | --- | --- | --- |
| Country (Spain vs UK) | 0.85 | 0.46 – 1.58 | 0.600 |
| Age group (per category increase) | 1.68 | 0.97 – 2.90 | 0.064 |
| Gender (Female vs Male) | 0.92 | 0.46 – 1.83 | 0.800 |
| Professional background (Secondary vs Primary) | 1.05 | 0.53 – 2.09 | 0.890 |
| Professional background (Other vs Primary) | 1.10 | 0.39 – 3.14 | 0.850 |
| Health service contacts (4–10 vs ≤3) | 1.15 | 0.64 – 2.07 | 0.650 |
| Health service contacts (≥11 vs ≤3) | 1.40 | 0.50 – 3.93 | 0.470 |
| Self-access (Yes vs No) | 4.74 | 1.80 – 12.53 | 0.002 |
| Country × Self-access interaction | 1.05 | 0.37 – 2.96 | 0.950 |

# Note. OR = odds ratio; CI = confidence interval. Reference categories: UK (country), Male (gender), Primary care (professional background), ≤3 service contacts (health service contacts), No self-access (record access).
